# Driving towards ecotechnologies

**DOI:** 10.1080/20477724.2018.1452844

**Published:** 2018-04-09

**Authors:** Devora A. Najjar, Avery M. Normandin, Elizabeth A. Strait, Kevin M. Esvelt

**Affiliations:** a Media Lab, Massachusetts Institute of Technology, Cambridge, MA, USA

**Keywords:** Gene drive, ecotechnology, community engagement, open science, malaria, Lyme, dengue

## Abstract

The prospect of using genetic methods to target vector, parasite, and reservoir species offers tremendous potential benefits to public health, but the use of genome editing to alter the shared environment will require special attention to public perception and community governance in order to benefit the world. Public skepticism combined with the media scrutiny of gene drive systems could easily derail unpopular projects entirely, especially given the potential for trade barriers to be raised against countries that employ self-propagating gene drives. Hence, open and community-guided development of thoughtfully chosen applications is not only the most ethical approach, but also the most likely to overcome the economic, social, and diplomatic barriers. Here we review current and past attempts to alter ecosystems using biological methods, identify key determinants of social acceptance, and chart a stepwise path for developers towards safe and widely supported use.

## Introduction

Ecological engineering technologies are unique amongst other biotechnologies because they will likely impact everyone living within the target region. Decisions to develop and deploy technologies with shared impacts are arguably matters of civic governance rather than the marketplace and regulation, a distinction with important implications for technology developers.

Recent advances involving gene drive systems in malarial mosquitoes have generated considerable excitement in the global health community. Gene drive systems can spread genetic changes through a wild population even when such changes reduce fitness. As first detailed by Austin Burt in 2003, gene drive systems can suppress populations by disrupting recessive fertility genes; suppressing a vector population could dramatically reduce transmission levels [1–3]. Alternatively, disease-resistance genes could be spread through a vector population, with a similar outcome. The advent of genome editing using RNA-guided CRISPR nucleases enables researchers to build gene drive systems that use CRISPR to cut the wild-type chromosome and replace it with an engineered counterpart in each generation of heterozygotes [4]. The versatility of CRISPR allows most genes in sexually reproducing species to be affected, while the ability to cut multiple sites theoretically renders CRISPR-based gene drive systems robust enough for use in the wild [5–7]. Functional proof-of-principle suppression and alteration gene drive systems have now been developed in An. gambiae and An. stephensi, respectively [8,9], and development is ongoing in other species.

Technical barriers to gene drive and other ecological engineering technologies are falling rapidly. With this in mind, scientists must consider how best to develop these and related technologies to navigate the social and political hurdles that may prevent them from benefiting the world. The earliest applications of a new technology will always be met with greater skepticism, with comparative successes or lack thereof impacting later developments. Because genetic engineering is already regarded with distrust and advances in gene drive are subjected to intense media scrutiny, there is a nontrivial likelihood of popular rejection and pressure to deny approval.

The pretext for rejection is likely to be diplomatic or economic. Even in countries for which international agreements such as the Cartagena Protocol governing transboundary movements are not applicable, the introduction of a transgenic product widely anticipated to cross borders would constitute a reason for other countries to justify erecting trade or transport barriers that may be desired for other reasons. The SPS agreement of the World Trade Organization covers restrictions designed ‘to protect human, animal or plant life or health … from risks arising from additives, contaminants, toxins or disease-causing organisms’ as well as ‘to prevent or limit other damage … from the entry, establishment or spread of pests,’ (SPS Agreement, Annex A, paragraph 1). As the agreement explicitly allows sanitary and phytosanitary measures intended to protect the health of fish and wild fauna, as well as of forests and wild flora, the potential entry of a gene drive system, especially one anticipated to spread indefinitely in the target species upon arrival, could easily be interpreted to apply. Notably, restrictions may be enacted even when scientific evidence remains insufficient. Given the potential for approval of beneficial releases to be legally denied, it seems prudent to engineer applications for popular support as well as technical effectiveness.

**Figure 1.**
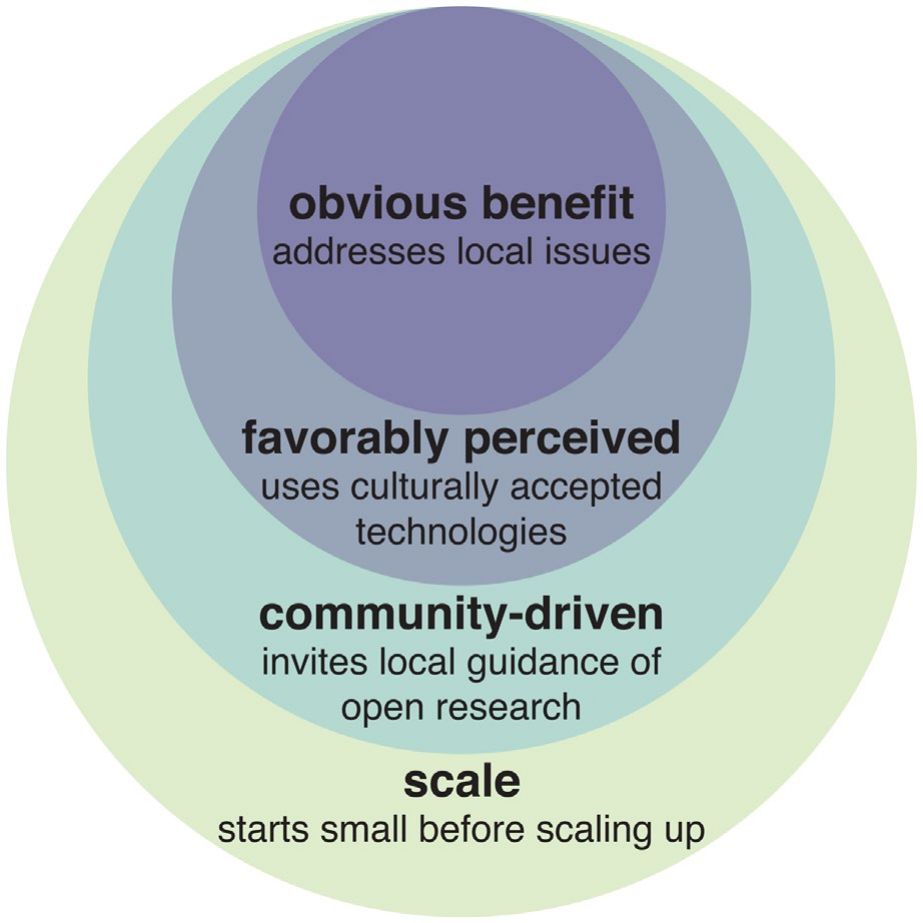
Key considerations for applications of ecotechnologies.

The single most important criterion for public support of a new technology is that the potential benefits of early proposals be obvious to typical citizens. Human health benefits are nearly always widely supported; applications aiming to improve the environment or animal welfare may also be acceptable in some cases. Improvements to agricultural efficiency might also be relevant for developing nations, but history suggests that these benefits may not be obvious to most citizens. While the specific challenges posed by each application will differ considerably, past and current examples of ecotechnologies highlight three additional key factors (Figure 1):

**Public perception**

Interventions perceived as being high-risk, even if arguably due to unjust negative associations, must offer additional benefits to earn support. In most cases, this will require addressing a serious and otherwise intractable ecological problem that is obvious to most citizens.

**Community involvement**

Early outreach and efforts to involve local citizens in making key decisions should begin at a project’s inception, with technology developers seeking to build community interest and support rather than focusing primarily on regulatory approval.

**Scale**

The more countries that will be impacted, the more difficult it will be to win support from the governments of all affected nations. This may become a problem for self-propagating gene drive systems that are likely to spread to most populations of a target species. Narrowing the scope of a project makes it easier to work with discrete communities and build local support while providing critical data for eventual applications elsewhere.

Taking account of these factors early in the technology development process can help ensure that ecological engineering applications are safe, supported, and beneficial.

## Public Perception: Choose (Favorably Perceived) Technologies

**Figure 2.**
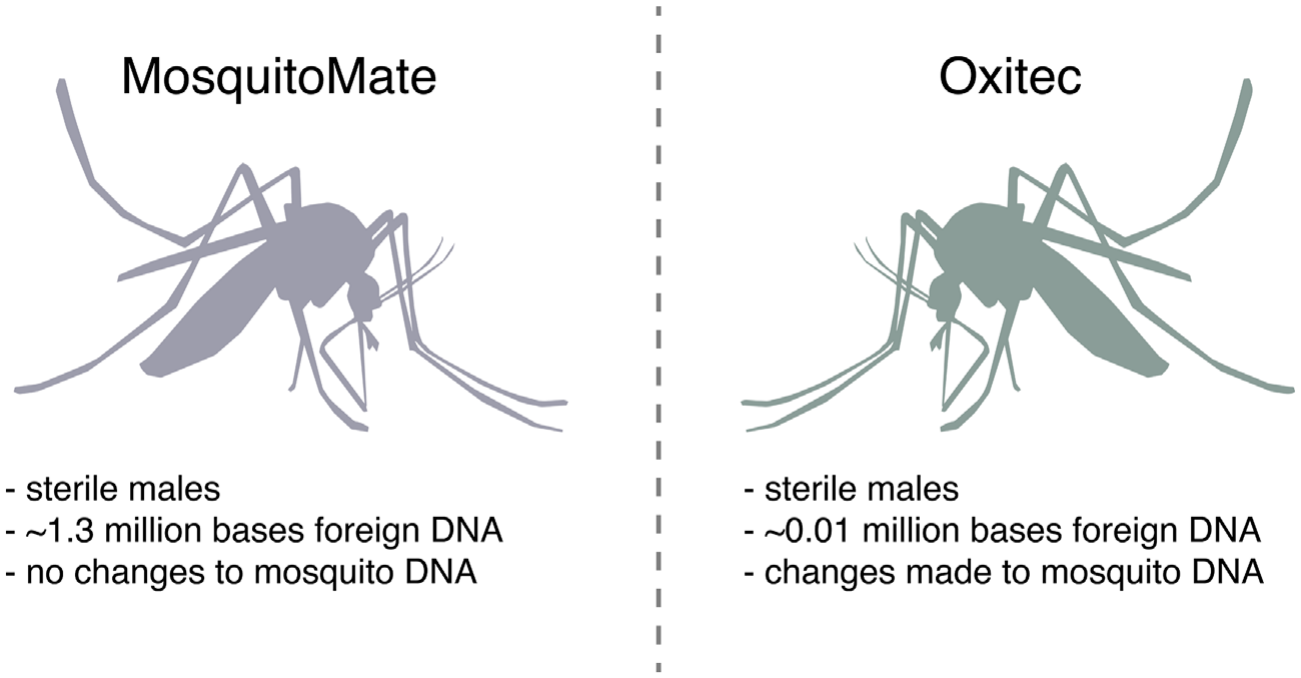
Public perception. The sterile males produced by MosquitoMate and Oxitec are functionally equivalent, but the difference in public perception was striking - possibly because artificial Wolbachia infection is perceived as more “natural” than editing the mosquito genome.

Fairly or not, many technologies enjoy a distinct advantage or suffer a penalty due to existing public opinion. The starkly differing public reactions to alternative methods of producing sterile male *Aedes aegypti* mosquitoes, which could suppress population levels and potentially reduce transmission of dengue, Zika, yellow fever, and chikungunya, is a case in point (Figure 2) [10].

**MosquitoMate**

One way to generate sterile males utilizes *Wolbachia*, a bacterium that resides in the cytoplasm of a plurality of insect species but is not naturally found in *Ae. aegypti* [11]. When a male infected with *Wolbachia* mates with a wild type uninfected female, it causes cytoplasmic incompatibility that renders all fertilized eggs nonviable [12,13]. Researchers at the University of Kentucky developed a strain of *Ae. aegypti* mosquito that is stably infected with *Wolbachia*, now produced by the for-profit MosquitoMate [14]. Importantly, the technique does not require any genetic changes to the mosquito or the *Wolbachia* genome.

Partly because it is viewed as more ‘natural’, this method of population suppression interested the Consolidated Mosquito Abatement District of Fresno and Clovis counties in central California, which has been working to eradicate *Ae. aegypti* since it appeared as an invasive species in 2013 [15]. In 2016, the District partnered with MosquitoMate to test their *Wolbachia*-infected sterile male mosquitoes in the Fresno area. Initial testing in summer 2016 under an EPA Experimental Use Permit saw roughly 40,000 sterile males released every week for ten weeks [16]. This year, MosquitoMate has teamed up with Verily, an Alphabet company, to scale up the number of sterile *Wolbachia* mosquitoes released in the Fresno area from thousands to millions per week and extended the program to 20 weeks [17]. There has been almost no public comment on the governmental approval of these field trials and the program has received positive support from the community [18–20].

**Oxitec**

The positive reception of MosquitoMate trials in Fresno and elsewhere in the U.S. starkly contrasts with the negative reception of a proposed Oxitec trial in the Florida Keys. Oxitec, a subsidiary of the bioengineering conglomerate Intrexon, engineered a transgenic *Ae. aegypti* mosquito with a proprietary self-limiting gene [21]. Unless exposed to tetracycline-class compounds during development, mosquitoes with the transgene will die before reaching adulthood [22]. Released male mosquitoes containing the transgene mate with wild females, producing offspring that will fail to develop and thereby suppressing the population.

Oxitec previously conducted several successful and popularly supported field trials and subsequent deployments of their mosquitoes in areas impacted by dengue throughout Central and South America as well as Malaysia [23–25]. The company was invited to conduct a U.S. trial by the Florida Keys Mosquito Control District Board of Commissioners [26]. Even after an FDA assessment issued a Finding of No Significant Impact for Oxitec mosquitoes in the Keys, local residents protested the release of genetically engineered mosquitoes into their ecosystem [27,28]. The FDA received over 2600 public comments on their approval of the field trial [18]. The Board of Commissioners subsequently placed a county-wide referendum on the issue on the general election ballot in November, 2016, the results of which showed mixed interest [29]. Though a slight majority of the larger Monroe County voted in favor, the suburb of Key Haven where testing would take place voted against the release of Oxitec mosquitoes, with a 65% majority opposed [30]. Though Oxitec has made attempts at community outreach and education, it evidently failed to convince a majority of residents, and the fate of the project is unknown [31,32].

Tellingly, the Florida Keys Mosquito Control District has since begun a collaboration with MosquitoMate and began conducting field trials in mid-April 2017 on Stock Island to determine whether its mosquitoes are effective at reducing the local population [33], with no outcry from the population. South Miami has also begun trials, with Mayor Philip Stoddard, a biology professor at Florida International University, commenting ‘It’s a lot less controversial because nothing has been genetically modified’ [34].

Given that the potential outcomes of both technologies are comparable, both companies are for-profit, and none of these areas has recently suffered from a serious dengue or Zika virus outbreak, the dramatic difference in reception was most likely due to pre-existing public views of the respective technologies. This is not to detract from Oxitec’s successes in areas where mosquito-borne disease is an obvious problem; scientifically, their method appears sound. But while the introduction of *Wolbachia* into *Aedes aegypti* is hardly natural, a preference for *Wolbachia*-infected sterile male mosquitoes rather than transgenic equivalents is consistent with widespread skepticism of genetic engineering: humans did not deliberately change any DNA sequences in *Wolbachia*-infected mosquitoes, even though they artificially introduced the entire *Wolbachia* bacterium, including its genome. Since community approval is integral to the success of eco-engineering applications, as is evidenced by the currently stalled attempt to introduce Oxitec mosquitoes in Florida, it is prudent to consider how different potential interventions will be perceived by the community before making substantial investments of time and money.

## Community involvement: early and extensive

**Figure 3.**
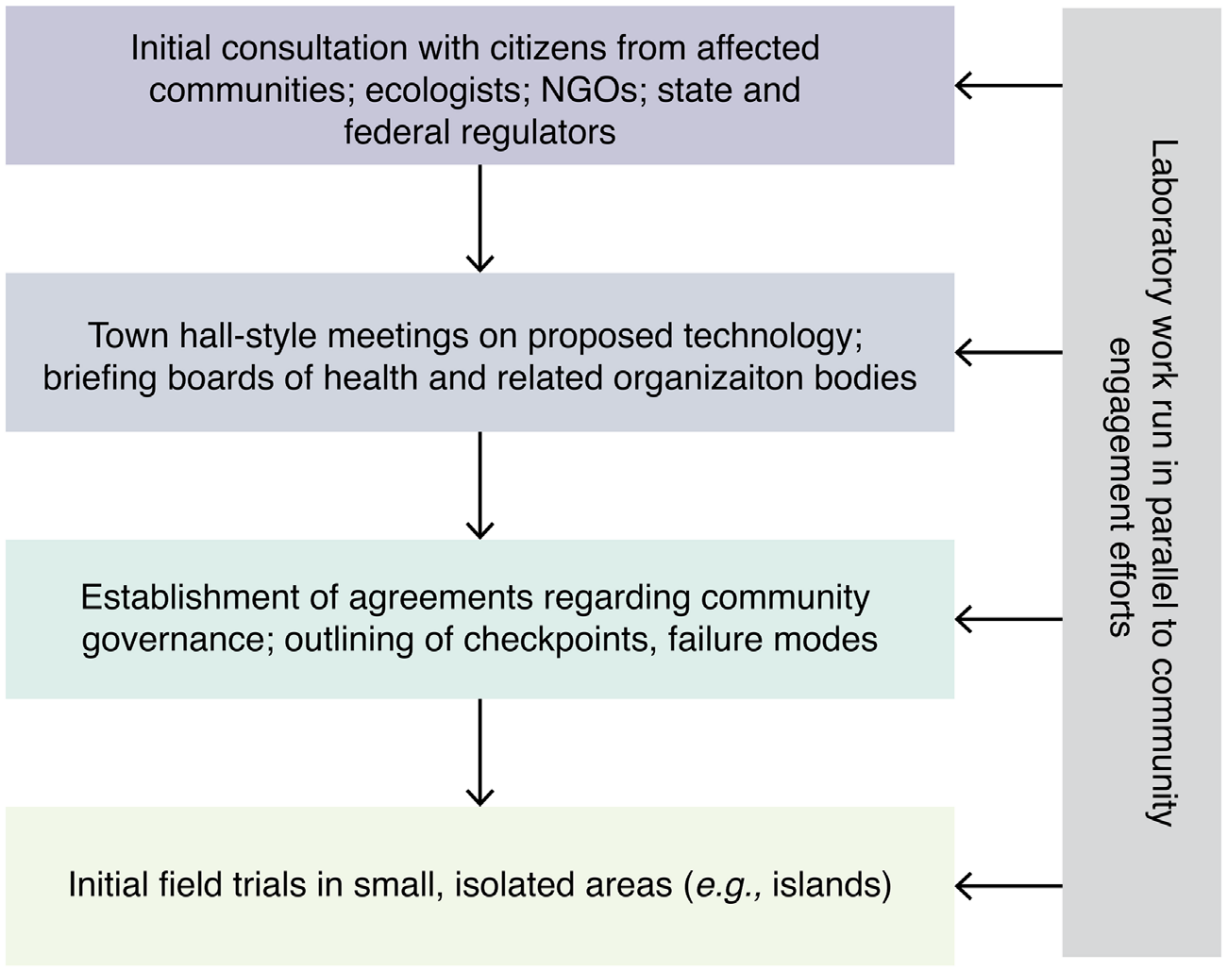
Community involvement. In the Responsive Science model, scientists engage longitudinally with communities and regulatory bodies. Ideally, the process should precede laboratory research.

Another difference between new ecological interventions and medical technologies is that local citizens are considerably more likely to be aware of useful ecological information to which researchers may not otherwise have access. Broadly speaking, science works by inviting others to challenge existing models; since local citizens may have uniquely relevant knowledge, the project will be safer and more effective if everyone is invited to participate. Ideally, the effort will become and be accurately viewed as one undertaken by the community as a whole (Figure 3).

**Eliminate Dengue**

An early example of successful community involvement occurred prior to the release of *Wolbachia*-infected *Ae. aegypti* in regions near Cairns, Australia. The effort was led by Scott O’Neill and colleagues, who began studying *Wolbachia* infection in the 1980s as a means of compromising vectorial transmission of dengue virus with a particular interest upon discovery of a particularly virulent strain of *Wolbachia* that halved the lifespan of fruit flies it infected in the early 2000s [35–39]. While reducing mosquito lifespan did not prove effective, the presence of less-virulent strains of *Wolbachia* inside the vectors was fortuitously discovered to interfere with viral replication and transmission in fruit flies. O’Neill quickly applied this finding to *Ae. aegypti* [40,41]. Preliminary caged trials discovered that *Wolbachia*-infected *Ae. aegypti* spread to fixation if released at sufficiently high densities, as expected [42], leading to creation of the nonprofit Eliminate Dengue.

Rather than work directly with distant populations in other countries, the Eliminate Dengue team chose to work with the local and comparatively highly educated communities near Cairns, Australia, where mosquitoes were endemic but the disease was rare. This neatly avoided concerns over initially testing a new technology in an area with a comparatively vulnerable population.

Eliminate Dengue took the unusual step of inviting the local community to share their concerns and criticism in order to guide safety testing before the technology was even ready for deployment. They went door to door explaining how infecting the local mosquito population with Wolbachia could prevent future dengue outbreaks and hosted numerous town hall meetings to publicly address community concerns and invite local citizen involvement [43,44]. By the time the project received approval from the Australian Pesticides and Veterinary Medicines Authority as well as a finding of negligible risk by the CSIRO, the team had won over more than 80% of citizens in the two Cairns towns being analyzed for a field trial and galvanized many community members to assist the project [45]. Citizens were given water, mosquito eggs, and food so that every household could raise and release their own *Wolbachia*-infected mosquitoes. Within a few months of beginning deployment, about 80–90% of mosquitoes carried *Wolbachia* [42]. When the mosquito population was reanalyzed a few years later, the mosquitoes were observed to have spread *Wolbachia* to descendants within the target areas [46].

Successful community involvement and deployment of Eliminate Dengue mosquitoes in the developed world laid the groundwork for subsequent work in communities at higher risk of disease. The project has grown from a small university-led effort to a major international nonprofit that partners with scientists and communities in countries from Southeast Asia to India to Brazil.

**Mice Against Ticks**

No ecological engineering project can or should move forward if fiercely opposed by the local community. Why not invite communities and their representatives to actively govern projects from inception by choosing among scientifically plausible development and testing options? In this model, scientists are advisors and technical hands, but not decision-makers.

Researchers at MIT and Tufts are assisting the local communities of Nantucket and Martha’s Vineyard (Massachusetts) with Mice Against Ticks, a project aiming to prevent Lyme disease, the most common vector-borne illness in the United States, and other tick-borne illnesses [47]. These islands have the highest per capita rates of probable and confirmed Lyme disease infections in Massachusetts and are among the most afflicted in the nation. According to the Board of Health, over 40% of Nantucket’s residents have been affected by tick-borne disease [48]. It is a problem that is manifestly obvious to everyone in the community, and one with few if any acceptable and effective solutions. Notably, island citizens are reluctant to eliminate or even to cull the abundant deer populations responsible for the dramatically increased number of ticks, and thus partly for the high number of human infections.

The white-footed mouse *Peromyscus leucopus* is the primary reservoir of the pathogens responsible for Lyme disease, babesiosis, anaplasmosis, ehrlichiosis, and Powassan, all of which are efficiently passed between mice and ticks [49,50]. Ticks become infected when they bite a mouse, then go on to bite and infect other mice, thereby increasing the size of the reservoir, as well as other organisms including humans. Researchers approached the communities with a proposal to address the problem by heritably immunizing the local populations of white-footed mice, which would remove the primary reservoir of disease and reduce the number of infected ticks [51,52].

If enacted, it would be the first time engineered organisms were released with the intention of lastingly altering the local wild population. Crucially, the mice could be immunized in several different ways, subjected to field trials examining effectiveness and unwanted side-effects on different (mostly uninhabited) islands, and released using one of several alternative approaches. All of these are decisions that could be made by local citizens, so the communities were asked to directly govern the project from inception.

According to majority community preference, all edited DNA will be native to the white-footed mouse - in this case antibody-encoding genes isolated from immunized *Peromyscus leucopus*. Also by current community preference, mice will be immunized against the Lyme disease pathogen *Borrelia burgdorferi* and also against the tick salivary protein subolesin, which disrupts transmission of all tick-borne diseases by causing ticks to drop off before completing a blood meal. The project obeys basic principles of engineering complex systems by way of solving the problem at hand through the smallest possible change, and starting small before scaling up.

Mice Against Ticks is now governed by Steering Committees appointed by the Boards of Health of each island following numerous town hall meetings; each committee includes a vocal skeptic who will ensure that all citizen concerns are heard and responded to. Remarkably, while many citizens outlined specific concerns that they wish to see addressed prior to any release, not a single voice has yet been raised in outright opposition to proceeding. Beyond their high disease burdens, the islands are noteworthy for the high fraction of well-educated citizens, strong local regulatory bodies, and traditions of town hall democracy. Whether the responsive science approach will translate to other communities–and whether the the islands will remain supportive once engineered organisms are no longer merely theoretical–remains to be seen.

## Scale: start small before scaling up

**Figure 4.**
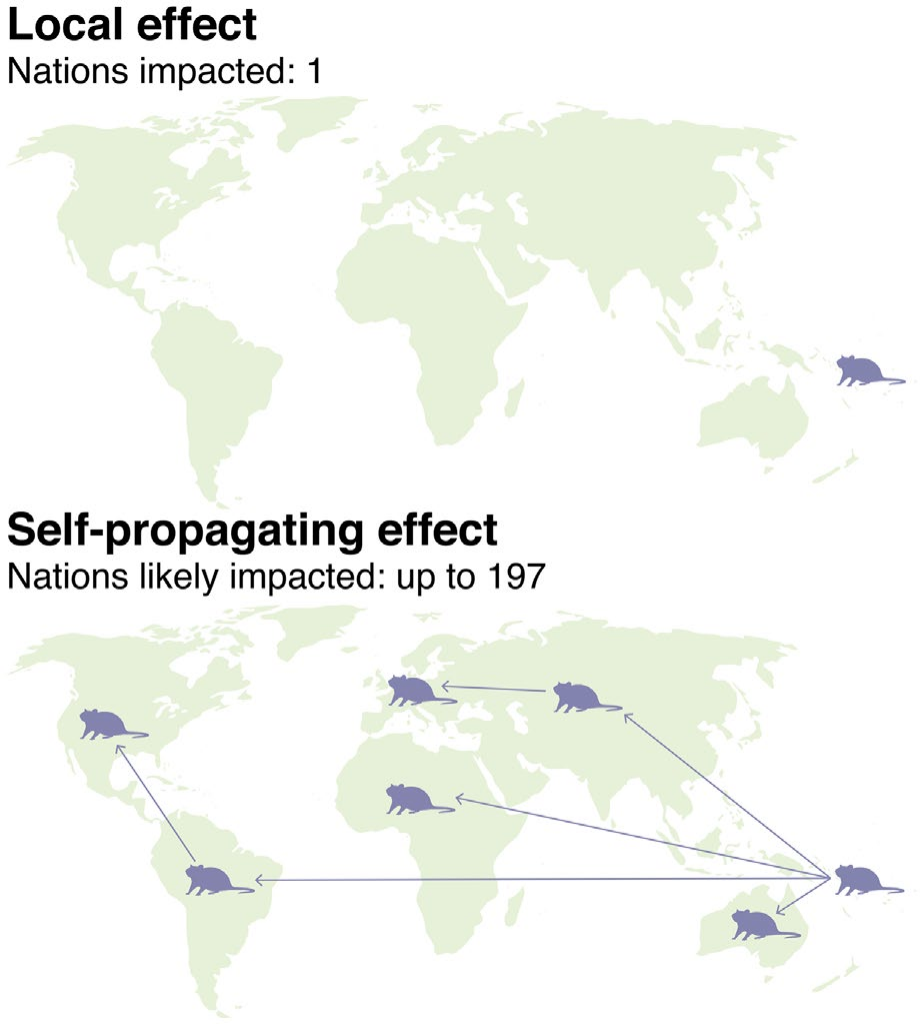
Scale. Local interventions can be developed in collaboration with early adopter communities and may be released after receiving regulatory approval from a single country. Interventions anticipated to spread without limit on their own are difficult to safely test at small scale and can face diplomatic complications given the likelihood of international spread.

When engineering a complex ecosystem, it is prudent to test proposed interventions at the local level before scaling up. It is easier to work with one or more local communities via face-to-face meetings, and simpler to obtain regulatory approval from one country than from several (Figure 4). Technologies likely to spread beyond a release site and cross international borders are far more complicated.

**Calicivirus release**

There are few if any historical parallels for a self-propagating gene drive system, but the closest is arguably the subset of biocontrol activities in which pathogens, parasites, or predators of an invasive species are introduced into the invaded environment. Unlike a self-propagating gene drive, biocontrol agents are unlikely to spread extensively and uncontrollably once introduced, but because this outcome is possible, field trials are often conducted on isolated islands. For example, rabbit calicivirus, the cause of Rabbit Haemorrhagic Disease (RHD), was first imported by Australia in 1991 to investigate its potential to control invasive rabbits [53]. The virus was initially tested on Wardang Island, where the myxoma virus had been tested for the same purpose four decades previously prior to its successful deployment on the mainland. But in September of 1995, RHD escaped to the mainland Australia, possibly carried by bush flies or blowflies on the wind, and rapidly spread across the continent [54]. In 1996, New Zealand, which also suffers from invasive rabbits, denied an application to import the virus. In response, a group of farmers illegally smuggled it in the following year through what is arguably the tightest biocontrol in the world [55].

Because the virus was released accidentally or illegally in the two countries, scientists were not able to orchestrate a controlled release to maximize effectiveness. Eventually, less-pathogenic strains of the virus evolved, as did resistance among the rabbits, greatly attenuating its ability to control the invasive population [53,56].

One important lesson of RHD is that even agents anticipated to spread on their own, including gene drive systems, are most effective when deployed according to a plan that takes weather, mating seasons, vectors, and other ecological variables into consideration. Post-release monitoring can identify problems such as resistance or poor spread and use this information to target subsequent waves of releases.

Perhaps more importantly, the story of RHD emphasizes that ‘controlled’ field trials are exceedingly difficult to design for interventions anticipated to spread indefinitely on their own. There is always a risk of the organism being released accidentally, as occurred in Australia, and once released, it may not be possible to subsequently bring the agent under control. Just as importantly, potential economic beneficiaries can deliberately transport the agent to areas it may not have been able to reach naturally, as occurred in New Zealand. The risk is demonstrable even when the system being tested is not well characterized and spreading the agent constitutes a legal offense. Worse still, self-propagating gene drive systems are unlike biocontrol agents in being unavailable elsewhere in the world. Given these complications, researchers considering whether to develop ecotechnologies with these characteristics, such as self-propagating gene drive systems, should carefully consider whether alternative methods may suffice.

**Target Malaria**

The lessons of RHD are perhaps most acute for Target Malaria, a nonprofit research consortium funded by the Bill and Melinda Gates Foundation and the Open Philanthropy Project that aims to contribute to the reduction and ultimate eradication of malaria in Africa. Because an effective vaccine arguably remains a distant prospect, using gene drive systems to suppress populations of Anopheles gambiae, Anopheles coluzzii, and Anopheles arabiensis may be the best hope of eradicating a disease that infected over 200 million people and claimed an estimated 429,000 lives in 2016 [57].

Researchers from Target Malaria are working to build two different kinds of population suppression gene drive systems. The first disrupts a recessive female fertility gene, which would cause populations to crash once the drive system becomes abundant [8]. The second distorts the sex ratio in favor of males by shredding the female-determining X chromosome during male meiosis, ultimately causing population collapse [58,59]. Both drive systems would be self-propagating, meaning that they are anticipated to spread on their own to most or all populations of the target species across sub-Saharan Africa.

While self-propagating interventions suffer from formidable social and diplomatic disadvantages, it is highly unlikely that any other approach can aspire to eradicate malaria. The major mosquito disease vectors in Africa are present across such large geographic areas, often with poor infrastructure, that the ability of a gene drive to spread indefinitely on its own would be vital for logistical reasons.

Self-propagating gene drive systems are not a silver bullet, not least because the natural spread of these systems would be quite slow: models suggest that natural spread from a single release would require over a decade to affect a mid-sized African country [3] (P. Welkhoff, personal communication). Effective deployment will require a systematic plan for geographically distributed releases across the entire target area, in combination with bed nets, insecticide spraying, antimalarial treatment and other control measures.

Moreover, releasing a single gene drive system may not be enough, as it is likely that some form of resistance will evolve. This is unlikely to be caused by mutations that block cutting, which models predict can be reliably overcome by targeting multiple sequences, but resistance arising from interference with with the nuclease or the guide RNAs, their expression, or some unexpected avenue are all within the realm of possibility [5]. We suspect that once the mechanism of a given form of resistance is identified, the targeting flexibility of CRISPR will permit the construction of new versions of the drive system that can overcome it by destroying the responsible genes at fertilization. But even if this proves to be the case, the new version will require even more time to spread, further necessitating a coordinated release plan. In short, every country harboring the target mosquitoes will likely need to approve on-the-ground support to achieve eradication, a potentially diplomatically challenging situation.

It is doubtful whether field trials of the self-propagating drive system can be conducted without a substantial risk of unintended spread. Models suggest that very few organisms need be introduced for drive systems of this type to invade a new population, meaning that any application effective enough to accomplish the intended outcome would be quite likely to (slowly) spread to most populations of the target species [60,61]. Given the horrific impact of malaria, people are highly likely to deliberately spread the drive system, even if illegal. Since unauthorized spread across international borders may result in one or more governments declining to cooperate with the eradication plan in reaction to the loss of sovereignty, identifying a path towards acceptance is crucial.

The Target Malaria team has been laying the groundwork for a decade, partnering with universities and research centers to build or renovate insectaries for mosquito research in Mali, Burkina Faso, and Uganda. These insectaries, which are supported by the Bill and Melinda Gates Foundation and the Open Philanthropy Project, undertake local scientific training programs and outreach, including public visits to the laboratories. Similar efforts will be needed in other nations, particularly to recruit local scientists who will be responsible for the majority of the project: deployment and monitoring.

To assess the ecological effects of suppressing mosquito populations and build confidence in local capabilities without the risk posed by a self-propagating gene drive, the initial mosquito strains imported and released will be sterile male lines. These may be followed by autosomal X-shredder strains or other local drive systems. A critical question is whether African communities will consider field trials using these alternatives to be adequate substitutes for the self-propagating gene drive system [62] that will likely be necessary to eradicate malaria. The mechanism may or may not be different, but the outcome and ecological ramifications will be the same: fewer mosquitoes.

## Implications for other applications

**Figure 5.**
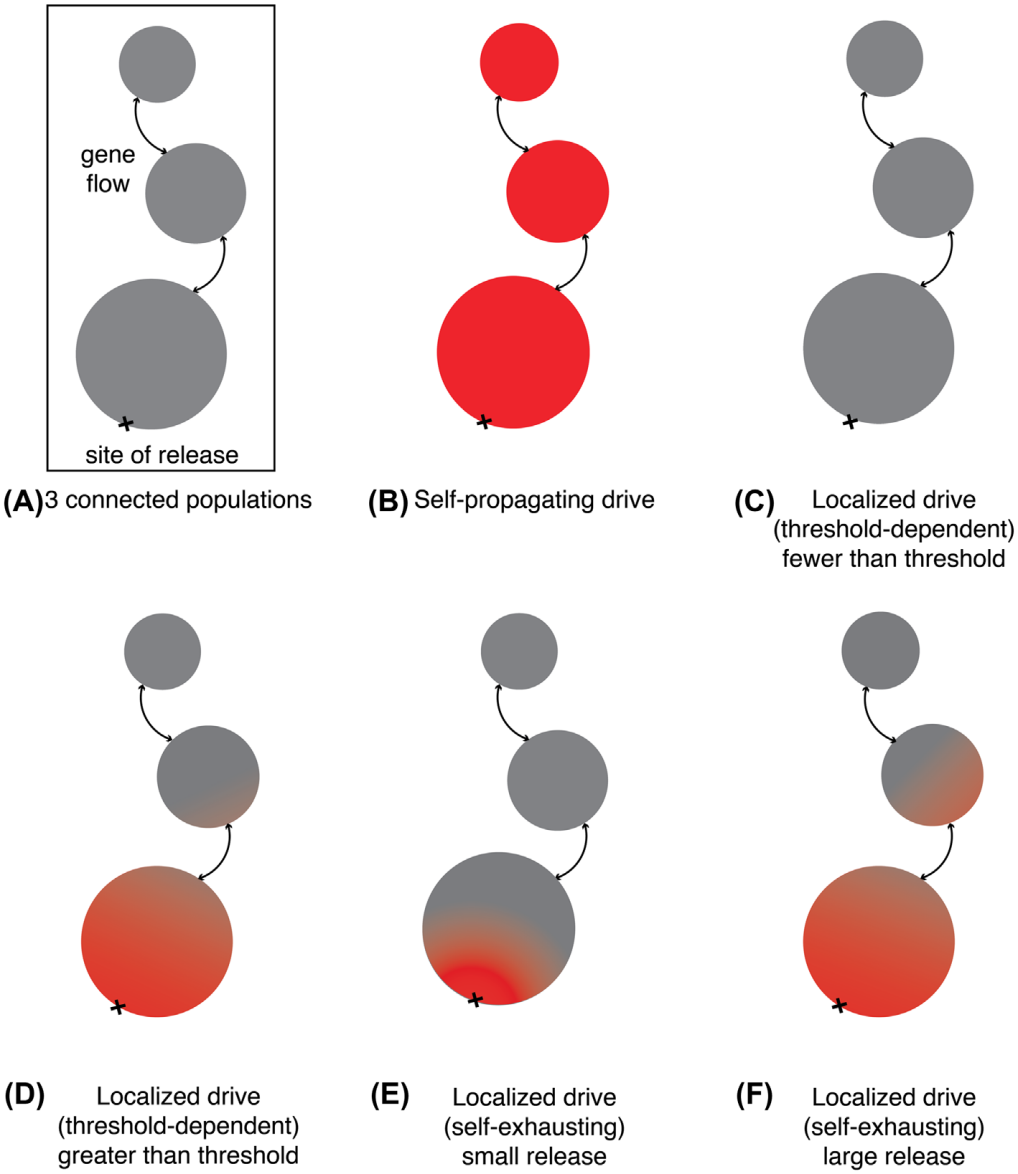
Geographic impacts of different gene drive types. (A) A depiction of the gene flow from a site of release through three interconnected geographic populations. (B) A self-propagating gene drive. (C) A threshold-dependent drive released at a frequency below the threshold. (D) A threshold-dependent drive released at a frequency above the threshold. (E) A self-exhausting drive released at comparatively low frequency. (F) A self-exhausting drive released at higher frequency. All depictions are approximate as exact values depend on drive efficiency, fitness cost, and other parameters.

Collectively, these existing ecological engineering programs offer lessons for researchers in choosing appropriate technologies for the problem at hand, and particularly for gene drive systems. Below are three classes of gene drive technology along with strengths and weaknesses (Figure 5, Table 1).

**Table 1.** Different classes of gene drive systems, the expected extent of spread, and applications.

Type of drive system	Anticipated spread	Best for
Self-Propagating	Can spread to most populations of the target species given gene flow	Time-sensitive, large-scale ecological issues with few alternative solutions (e.g. malaria)
Threshold-Dependent	Can spread within local populations if released above a given threshold; limits gene flow	Local ecological problems where it is acceptable to release large number of organisms and gene flow is a problem
Self-Exhausting	Can spread transiently within local and adjacent populations to an extent determined by gene flow	Local ecological problems where few organisms can be released and gene flow is not a problem
Self-Exhausting + Threshold	Can spread transiently within a local population and persists only if it exceeds a threshold frequency; limits gene flow	Community-by-community decision making

**Standard: self-propagating gene drive**

Most discussions of gene drive refer to self-propagating CRISPR-based drive systems. Releasing just a few organisms carrying a self-propagating gene drive system into a wild population could eventually affect most populations around the world [61]. Only a handful of large-scale problems, most notably malaria eradication, may both require the power of a standard self-propagating gene drive and be serious enough to earn sufficient popular support without the benefit of a field trial. The elimination of New World screwworm and the eradication of schistosomiasis are other possible applications. Even in these cases, methods of duplicating the intended ecological effect at a local scale may be useful in order to evaluate ecological effects [62], particularly since self-propagating gene drives are the most likely to allow other countries to credibly threaten trade barriers against a nation considering deployment. Scientists should think carefully on whether a self-propagating drive system is absolutely required to solve the problem at hand before initiating research.

**Local: threshold-dependent drive**

A threshold-dependent gene drive system increases in frequency only when the number of drive organisms is above a threshold level in the local population, and otherwise declines (Figure 5(c and d)) [62,63]. Examples include underdominance generated by toxin-antitoxin systems [64,65], chromosomal translocations [66], or swapped haploinsufficient genes [67]. Because they can be removed from a population by reducing the local frequency below the threshold, they benefit from the favorable perception of reversibility. Threshold-dependent drive systems might be used to keep effects confined within the borders of supportive communities [67]. Especially if constructed without inserting foreign DNA, they offer many social advantages. The only negative is the greater number of organisms that must be released.

**Local: self-exhausting drive**

Releasing a small number of organisms carrying a self-exhausting drive can impact a substantial fraction of the local population, but unlike a self-propagating drive, the effects will not spread indefinitely (Figure 5(e and f)). Examples include killer-rescue [68] and daisy drive [69,70], the latter of which can accomplish population suppression [69,71]. Typically requiring fewer organisms to be released than most threshold-dependent drives, self-exhausting drives are theoretically temporary, which in some circumstances may be an advantage, but suffer from their inability to impede gene flow into neighboring populations. They may be useful when it is impractical to release large numbers of organisms or as field trials for other types of drive systems. Self-exhausting drive systems that become threshold-dependent once exhausted would be particularly useful for efficiently altering local populations while also minimizing gene flow across political boundaries.

**Non-driving alternatives**

Genetic elements that do not exhibit drive, including Y-linked elements that impede female fertility [71], autosomal X-shredders, RIDL, and equivalents are not gene drive systems and have been reviewed extensively elsewhere [72], but the same social and economic considerations are relevant. Notably, the absence of gene drive does not prevent gene flow across boundaries, meaning that non-sterile constructs may face greater barriers than threshold drive systems.

## Conclusion

Scientists and technologists often focus exclusively on technical and regulatory concerns when developing new technologies. Because ecological engineering necessarily impacts all individuals within affected environments, this traditional approach is an ethical and practical mistake: social and economic factors are equally likely to determine whether a given technology will be successful. History suggests that if early applications address obvious problems, are aligned with local value systems, openly invite community guidance from project inception, and start at the local level before scaling up, the results are considerably more likely to benefit humanity and the natural world.

## Funding

The authors are grateful for support from Burroughs Wellcome IRSA 1016432 (to K.M.E.), DARPA Safe Genes N66001-17-2-4054 (to K.M.E.), NIH DP2 New Innovator AI136597-01 (to K.M.E.), and a Greenwall Foundation ‘Making a Difference’ award (to K.M.E.). The funders had no role in preparation of the manuscript or decision to publish.

## Disclosure statement

K.M.E. is an inventor on patents filed by MIT and Harvard University concerning various forms of self-propagating, self-exhausting, and threshold-dependent drive systems.
